# Pharmacokinetics and Efficacy of PEGylated Liposomal Doxorubicin in an Intracranial Model of Breast Cancer

**DOI:** 10.1371/journal.pone.0061359

**Published:** 2013-05-01

**Authors:** Carey K. Anders, Barbara Adamo, Olga Karginova, Allison M. Deal, Sumit Rawal, David Darr, Allison Schorzman, Charlene Santos, Ryan Bash, Tal Kafri, Lisa Carey, C. Ryan Miller, Charles M. Perou, Norman Sharpless, William C. Zamboni

**Affiliations:** 1 Department of Medicine, University of North Carolina at Chapel Hill, Chapel Hill, North Carolina, United States of America; 2 Lineberger Comprehensive Cancer Center, Chapel Hill, North Carolina, United States of America; 3 Department of Human Pathology, Integrated Therapies in Oncology Unit, University of Messina, Messina, Italy; 4 Department of Biostatistics, Lineberger Comprehensive Cancer Center, Chapel Hill, North Carolina, United States of America; 5 Division of Pharmacotherapy and Experimental Therapeutics, Eshelman School of Pharmacy, University of North Carolina, Chapel Hill, North Carolina, United States of America; 6 Translational Oncology and Nanoparticle Drug Development Initiative (TOND2I) Lab, University of North Carolina at Chapel Hill, Chapel Hill, North Carolina, United States of America; 7 Gene Therapy Center and Department of Microbiology and Immnunology, University of North Carolina at Chapel Hill, Chapel Hill, North Carolina, United States of America; 8 Department of Pathology and Laboratory Medicine, University of North Carolina, Chapel Hill, North Carolina, United States of America; 9 Department of Genetics, University of North Carolina, Chapel Hill, North Carolina, United States of America; 10 Carolina Center of Cancer Nanotechnology Excellence, University of North Carolina at Chapel Hill, Chapel Hill, North Carolina, United States of America; 11 Institute of Pharmacogenomics and Individualized Therapy, University of North Carolina at Chapel Hill, Chapel Hill, North Carolina, United States of America; Wayne State University School of Medicine, United States of America

## Abstract

**Introduction:**

Breast cancer brain metastases (BCBM) are a challenging consequence of advanced BC. Nanoparticle agents, including liposomes, have shown enhanced delivery to solid tumors and brain. We compared pharmacokinetics (PK) and efficacy of PEGylated liposomal doxorubicin (PLD) with non-liposomal doxorubicin (NonL-doxo) in an intracranial model of BC.

**Methods:**

Athymic mice were inoculated intracerebrally with MDA-MB-231-BR-luciferase-expressing cells. Tumor-bearing mice were administered PLD or NonL-doxo at 6mg/kg IV×1 and were euthanized prior to and 0.083, 1, 3, 6, 24, 72 and 96 h post-treatment. Samples were processed to measure sum total doxorubicin via HPLC. PLD and NonL-doxo were administered IV weekly as single agents (6 mg/kg) or in combination (4.5 mg/kg) with the PARP inhibitor, ABT-888, PO 25 mg/kg/day. Efficacy was assessed by survival and bioluminescence.

**Results:**

Treatment with PLD resulted in approximately 1,500-fold higher plasma and 20-fold higher intracranial tumor sum total doxorubicin AUC compared with NonL-doxo. PLD was detected at 96 h; NonL-doxo was undetectable after 24 h in plasma and tumor. Median survival of PLD-treated animals was 32 days (d, [CI] 31–38), which was significantly longer than controls (26d [CI 25–28]; p = 0.0012) or NonL-doxo treatment (23.5d [CI 18–28], p = 0.0002). Combination treatment with PLD/ABT-888 yielded improved survival compared to NonL-doxo/ABT-888 (35d [CI 31–38] versus 29.5d [CI 25–34]; p = 0.006).

**Conclusions:**

PLD provides both PK and efficacy advantage over NonL-doxo in the treatment of an *in vivo* model of BCBM. The results provide preclinical rationale to translate findings into early phase trials of PLD, with or without ABT-888, for patients with BCBM.

## Introduction

Brain metastases arising from breast cancer are a burgeoning clinical problem associated with decline in quality of life, loss of independence, and poor survival [1]. The incidence of brain metastases is highly subtype-dependent [2] such that patients with triple negative and Her2-positive advanced breast cancer are at highest risk for intracranial recurrence [3], [4]. Moreover, prognosis following the development of brain metastases is also associated with breast cancer subtype where survival following central nervous system recurrence is 3 to 4 months for women with triple-negative disease compared to 9 and 15 months for HER2-positive and endocrine-sensitive counterparts, respectively [5]. The current treatment paradigm for breast cancer brain metastases (BCBM) across all subtypes includes radiation therapy (whole brain and/or focused brain radiation) and/or surgical resection [6]. Although studies illustrate systemic therapy sequenced after cranial radiation improves outcome for many patients with breast cancer brain metastases, the physical properties of the blood brain barrier and the relative paucity of targeted agents to treat intracranial breast cancer remains a significant challenge in the development of systemic therapies capable of controlling both intra- and extracranial advanced breast cancer [7], [8], [9].

The development of chemotherapeutic agents to effectively treat solid tumors within or outside of the central nervous system depends, in part, on the ability of these agents to achieve cytotoxic drug exposure within the tumor(s). Encapsulating common anti-cancer agents into nanoparticle delivery systems, particularly liposomes, provides a promising approach to enhance central nervous system delivery. Although the mechanism of enhanced brain delivery is not completely understood, it is postulated that the higher exposure to central nervous system tumors is related to longevity in blood and altered distribution compared to non-nanoparticle, standard, small molecule formulations [10], [11], [12]. Prolonged systemic exposure afforded by nanoparticle technology may allow for permeation of tumor microcirculation via passive convection transport through a blood brain barrier potentially “compromised” by the presence of tumor [10], [11], [12]. Chances for extravasation improve with prolonged circulation half-life and a greater number of circulation passages through a tumor bed. Although factors inherent to intracranial tumor (i.e. increased intracranial pressure) may dampen the effect of nanoparticle transport into a tumor compartment, results of prior preclinical and clinical studies argue that longer circulation time afforded by nanoparticle formulations may overcome these effects [12], [13], [14]. However, the benefit of nanoparticle anti-cancer agents, with or without targeted agents capable of crossing the blood brain barrier, has yet to be fully examined in an *in vivo* model system of intracranial breast cancer.

In the current study, we utilized an intracranial model of aggressive triple negative breast cancer to evaluate the pharmacologic disposition and activity of a chemotherapeutic agent that is highly active in the treatment of breast cancer, namely the anthracycline doxorubicin [15], in a PEGylated liposome (PLD) formulation as compared to non-liposomal doxorubicin (NonL-doxo). To assess the efficacy of this approach, survival following treatment with PLD was compared to treatment with NonL-doxo. Finally, ABT-888, an inhibitor of a poly (ADP-ribose) polymerase [PARP] and subsequent DNA repair, has been shown to cross the blood brain barrier [16]. Thus, we sought to augment intracranial efficacy by combining PLD, a DNA-damaging agent which intercalates between base pairs of the DNA/RNA strand, thus preventing macromolecular biosynthesis [17], with ABT-888 in an intracranial model of breast cancer.

## Materials and Methods

### Ethics statement

All animal studies were conducted in accordance within the guidelines of the Guide for the Care and Use of Laboratory Animals of the National Institutes of Health and with the approval of the University of North Carolina at Chapel Hill's Institutional Animal Care and Use Committee (IACUC) on protocols 09–151 and 10–230.

### Cell lines and culture conditions

The MDA-MB-231-BR cell line was selected for study and was kindly provided as a gift by Toshiyuki Yoneda, PhD (The University of Texas Health Science Center of San Antonio)[18]. The MDA-MB-231-BR cell line and its parental cell line (MDA-MB-231) were derived from a metastatic pleural effusion of a 51-year old white female patient. The MDA-MB-231-BR cell line is a ‘brain-seeking’ subclone that, following serial *in vivo* and *in vitro* selection, more frequently metastasizes to the brain in preclinical models versus its parental line [18]. The MDA-MB-231-BR cell line was cultured in DMEM (Life Technologies, Grand Island, NY) with 10% FBS (fetal bovine serum; Sigma-Aldrich, St. Louis, MO). The cell line was grown with penicillin/streptomycin (Life Technologies, Grand Island, NY) at 37°C and 5% carbon dioxide. Cells were harvested immediately prior to intracranial implantation. In addition, the identity of the MDA-MB-231-BR cell line was confirmed by global gene expression analyses (September 2010).

### Luciferase transduction of MDA-MB-231-BR

The MDA-MB-231-BR cell line was transduced with the bicistronic lentiviral vector pTK1261 carrying the firefly Luciferase under the control of a CMV promoter and a fusion green fluoresence protein/Blasticidin (PFG/BSD) under translational control of an IRES. All lentiviral vectors were prepared by using the calcium phosphate method to transiently transfect 293T cells with 15 μg vector plasmid, 10 μg packaging plasmid, and 5 μg envelope plasmid [19]. All vectors were pseudotyped with the VSV-G envelope protein. Luciferase-expressing vector concentrations were determined by p24*^gag^* ELISA.

### Pharmacologic agents

Doxorubicin (NonL-doxo) and PEGylated liposomal doxorubicin (PLD, Doxil®) were both obtained from the University of North Carolina (UNC) Hospitals Pharmacy (Chapel Hill, NC). The poly-ADP-ribose polymerase (PARP) inhibitor, ABT-888, was synthesized by the Chemistry Center for Integrative Chemical Biology and Drug Discovery at UNC (Dr. S. Frye). ABT-888 was dissolved in PBS (phosphate-buffered saline, Life Technologies, Grand Island, NY). The molecular weight of pure ABT-888 is 244.29 g/mol, while the molecular weight of the salt form (which includes 2 HCl molecules) is 317.21 g/mol. All calculations were performed using the molecular weight of the salt form of ABT-888. The ability of ABT-888 to inhibit the formation of PAR was confirmed by measuring PAR levels in MDA-MB-231-BR cellular extracts using HT PARP *in vivo* Pharmacodynamic Assay II (Trevigen, Gaitherburg, MD, data not shown).

### Cell viability assay

A mitochondrial dye conversion assay (Cell Titer 96® Aqueous One Solution Cell Proliferation Assay; Promega Corp., Madison, WI) was used to measure cell viability after treatment. This assay was conducted according to manufacturer's instructions, with modifications. Briefly, 10,000 MDA-MB-231-BR cells were seeded in each well of a 96-well plate. Cell counting was performed with an automated cell counter. Cells were allowed to adhere overnight, and media was replaced with fresh media containing a range of drug doses. After the specified treatment period, 20 µl of tetrazolium compound inner salt (MTS) containing an electron coupling reagent, phenazine ethosulfate (PES) was added in each well and incubated at 37°C for 1 hour (h). The MTS absorbance was measured spectrophotometrically at 490 nm (minus background absorbance). All cytotoxic experiments were done in triplicate.

### Single agent and combination treatments in cell lines

The IC50, defined as the inhibitory concentration that caused a 50% reduction in MTS dye conversion (i.e. 50% *in vitro* response inhibition), was defined for both ABT-888 and NonL-doxo after 72 h of treatment. Of note, PLD was not tested *in vitro* as the goal of our study was to evaluate the differences in PK and efficacy of PLD vs. NonL-doxo in an *in vivo* environment. Drug combination interactions were analyzed using methods developed by Chou and Talalay [20]. As per prior work [21], the following treatment schedules were tested: 1) 72 hours (h) NonL-doxo followed by 72 h ABT-888, 2) 72 h ABT-888 followed by 72 h NonL-doxo, and 3) 72 h concurrent schedule of NonL-doxo and ABT-888 in combination. Using cell lines plated as described above, treatment combinations consisting of a constant ratio of 308/1 (ABT888 [uM]/NonL-doxo [uM]) were applied to cells and growth was measured compared to untreated controls using Cell Viability assay.

### Animal use and intracranial tumor inoculation

The mice were housed within a BSL2 facility and in sterile caging. Therapeutic studies and assessment for response were performed with the assistance of the Mouse Phase I Unit (MP1U). Female, *Foxn1^nu/nu^* mice, approximately 20 grams and aged 8 weeks of age were bred in-house from animals purchased from Charles River Laboratories International, Inc. (Wilmington, MA; Stock #490) and were used for all studies. Prior to intracerebral tumor inoculation, mice were anesthetized with ketamine 75 mg/kg IP ×1 and Domitor® 1mg/kg IP ×1. Mice were placed into a stereotactic frame (Kopf Model 900, Tujunga, CA) prior to injection of 2×10^5^ MDA-MB-231-BR cells in a volume of 5uL of 5% methylcellulose and culture media. Cells were stereotactically-injected into the right caudate nucleus of the basal ganglia using a 27 gauge needle (from Bregma a:1.0mm, l:-2.0 mm, d:-4.0 mm) which remained in place for a period of 2 minutes (min) to minimize reflux through the needle track.

### Bioluminescence imaging

All animals were anesthetized via inhalation with 2% vaporized isoflurane during the imaging process. Approximately 15 min prior to imaging, all inoculated mice received an intraperitoneal (IP) injection of D-Luciferin dissolved in PBS (150 mg/kg; Caliper Life Sciences, Hopkinton, MA). To confirm the presence or absence of intracranial tumor, mice were imaged using the IVIS Lumina camera (Caliper Life Sciences, Hopkinton, MA). Images were analyzed using Living Image 4.0 Software (Caliper Life Sciences, Hopkinton, MA). All values were recorded as photons/second and were corrected for the presence of background signal.

### Pharmacokinetic study design

On Day 18 day following intracranial injections of 2×10^5^ MDA-MB-231-BR cells, mice were pair-matched into 2 treatment groups. Intracranial tumor formation was confirmed by serial bioluminescence imaging approximately 1 and 2 weeks post-injection. Prior to treatment, the average luciferase signal (photons/second) between groups was not statistically different (data not shown). Group 1 (n = 23) received NonL-doxo administered over 10–15 seconds at 6 mg/kg IV ×1 via tail vein. Group 2 (n = 23) received PLD administered over 10–15 seconds at 6 mg/kg (doxorubicin equivalents) IV ×1 via tail vein.

Mice (n = 3 per time point, n = 2 prior to administration) were sacrificed prior to administration, and at 0.083, 1, 3, 6, 24, 72 and 96 hours (h) after administration of NonL-doxo and PLD. Approximately 1 mL of blood was collected via terminal cardiac puncture following deep anesthesia (ketamine 100 mg/kg IP ×1 and Domitor® 1 mg/kg IP ×1) using sodium heparin as an anticoagulant and centrifuged at 1,500 g for 5 minutes to collect plasma. Brain tumor, peri-tumoral brain and normal (contralateral, non-tumor) brain were collected from each mouse. Plasma, tumor, and tissues were placed in cryopreservation vials and preserved by snap freezing using liquid nitrogen. All samples were stored at −80°C until analysis.

### Sample processing and analytical method

Total tissue and tumor weight was recorded at time of collection. Whole tissue and tumors were snap frozen in liquid nitrogen and stored at −80°C until homogenized. To form tissue and tumor homogenates, the intact tissues and tumors were thawed and sectioned [11]. The sections were weighed and diluted in a 1:3 ratio with phosphate buffered saline (PBS) solution. Finally, these mixtures were homogenized by placing zirconium oxide beads (15 small and 2 large; Omni International Inc, Kennesaw, GA) into 2 mL tubes at 3,000×*g* using a Precellys 24 homogenizer (Omni International Inc, Kennesaw, GA) twice for 15 sec each with a 5 sec wait between each run.

Samples were further processed for the analytical studies using protein precipitation with acetonitrile. An 800 µL of extraction solution containing internal standard (acetonitrile with 100 ng/mL daunorubicin) was added to 200 µL of plasma, tumor or tissue homogenate into a 2 mL microcentrifuge tube. The samples were vortexed for 10 min and centrifuged at 10,000×*g* for 10 min at 4°C. A 900 µL of the supernatant was decanted into clean tubes, evaporated under nitrogen in TurboVap and reconstituted in 150 µL of 25% acetonitrile solution (containing 0.026 M Na2HPO4, 3.5 mM triethylamine and pH adjusted to 4.6 with 3M citric acid). The samples were then vortexed, transferred into auto-sampler vials and analyzed by high-performance liquid chromatography (HPLC) using fluorescence (FL) detection (excitation wavelength: 490 nm/ emission wavelength: 590 nm). The HPLC-FL method to measure sum total (encapsulated and released) doxorubicin in plasma, tumor, and tissues was modified from previous studies [22], [23], [24].

### Efficacy study design

Mice with luciferase-confirmed intracranial tumor were placed into the following treatment groups 7 to 14 days following intracranial inoculation of MDA-MB-231-BR cells: 1) control, IV (via tail vein) PBS (100 uL) weekly, 2) NonL-doxo 6 mg/kg IV weekly, (3) PLD 6 mg/kg IV weekly, (4) ABT-888 25 mg/kg/day via oral gavage (OG), (5) NonL-doxo 4.5 mg/kg IV weekly plus ABT-888 OG 25 mg/kg/day, or (6) PLD 4.5 mg/kg IV weekly plus ABT-888 OG 25 mg/kg/day. Treatment was ongoing until clinical symptoms necessitated sacrifice. Prior to treatment, the average luciferase signal (photons/second) between groups and within each experiment was not statistically different (data not shown). Mice were weighed a minimum of three times/weekly and were monitored by optical imaging weekly until clinical symptoms (i.e. decreased response to stimuli, neurologic dysfunction, weight loss of 20% and/or a Body Composition Score of 2 or less) necessitated sacrifice.

### Statistical analysis

#### Cell line studies

CalcuSyn (BioSoft, Cambridge, UK) was used to estimate the IC50 for each drug and to determine the combination index, which is a measurement of the type of drug interactions. A combination index (CI) of 1 indicates an additive response, <1 indicates a synergistic response, and >1 indicates an antagonistic response.

#### Pharmacokinetic analysis

The pharmacokinetics of PLD and NonL-doxo in plasma, tumor and tissues were analyzed by noncompartmental analysis using WinNonlin Professional Edition version 5.2.1 (Pharsight Corp., Cary, NC). The area under the concentration versus time curve from 0 to ∞ (AUC_0–∞_) was calculated using the linear up/log down rule. The plasma volume of distribution (Vd), clearance (CL), and half-life (t_1/2_) were calculated using standard equations. The maximum concentration (C_max_) and time of C_max_ (t_max_) were determined by visual inspection of the concentration versus time data.

#### Efficacy analysis

The Kaplan-Meier method and Log-rank tests were used to evaluate and compare overall survival among treatment groups. Unadjusted p-values are reported. For bioluminescence imaging, fold changes were calculated relative to the start date of treatment. If present, negative imaging values (due to correction for background) were recorded and set to zero. For every time point where at least two animals were alive in the treatment group, the median level and interquartile range (25^th^–75^th^ percentile) for bioluminescence imaging were calculated.

## Results

Pharmacologic disposition and drug efficacy studies were conducted in a murine model of intracranial breast cancer. Briefly, 2×10^5^ MDA-MB-231-BR cells were stereotactically implanted into the right caudate nucleus of *Foxn1^nu/nu^* mice. Intracranial tumor formation was monitored *in vivo* via bioluminescence imaging (mean signal 4.5×10^6^, standard deviation 7.0×10^6^ photons/second approximately 7 days post-implantation). Liposomal and non-liposomal drugs were administered, and tumor and body tissue drug concentrations determined over time. The median survival of treated versus untreated animals (26 days, 95% Confidence Interval [CI] 25–28 days) was determined as an objective measure of efficacy.

### Pharmacokinetic study results: plasma, tissue and tumor pharmacokinetic disposition

#### Nonliposomal Doxorubicin (NonL-doxo)

**Figure 1:**
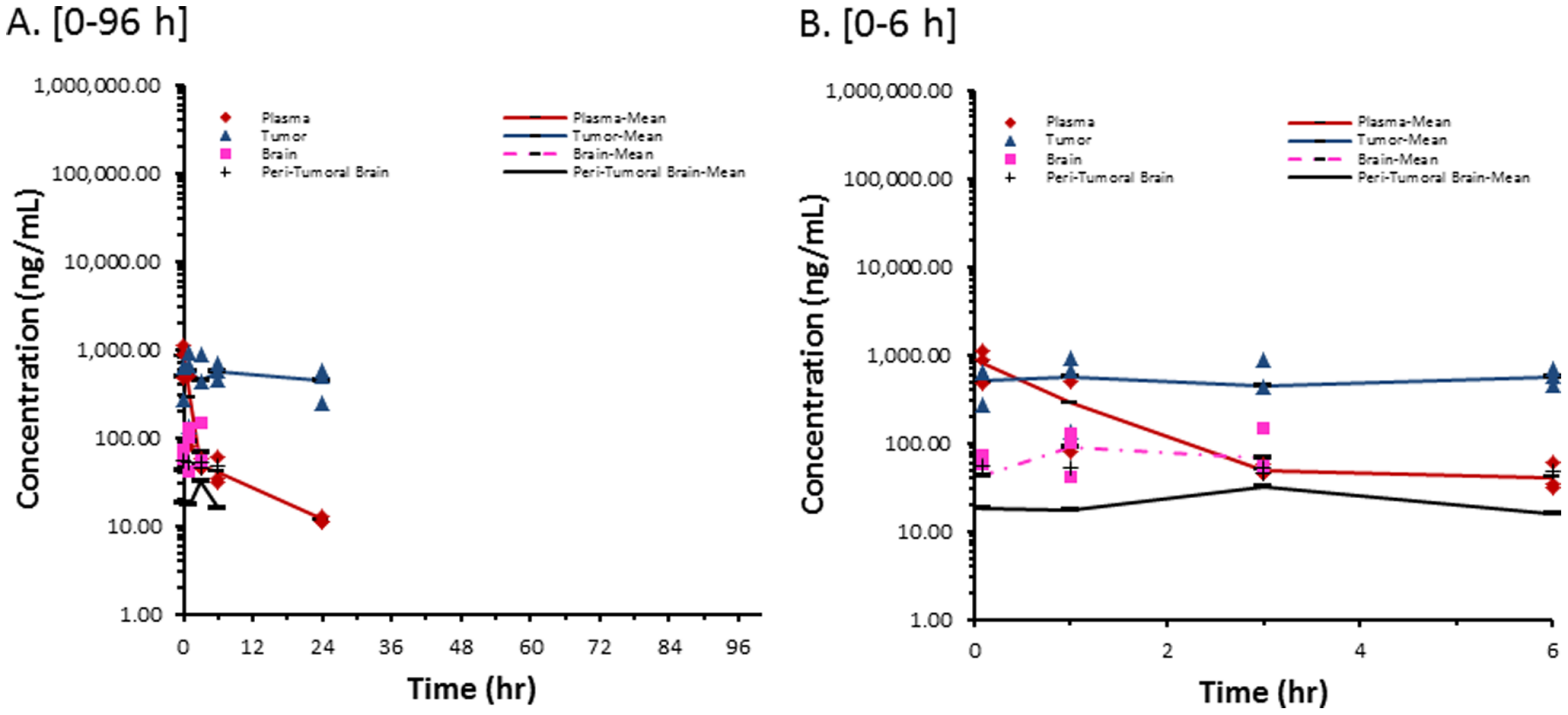
Sum Total Doxorubicin Concentrations from NonL-doxo. Individual and mean sum total doxorubicin concentration in plasma, brain tumor, contralateral non-tumor brain, and peri-tumoral brain of female athymic nude (nu/nu) mice bearing intracranial MDA-MB-231-BR human triple-negative breast cancer xenografts following administration of nonliposomal doxorubicin (NonL-doxo) at 6 mg/kg IV ×1. Samples (n = 3 mice at each time point) were obtained at 0.083, 1, 3, 6, 24, 72 and 96 hours following administration of NonL-doxo. Concentrations were undetectable after 3 hours (contralateral non-tumor brain), 6 hours (peri-tumoral brain), and 24 hours (plasma and tumor) of administration. (**A**) 0–96 h; (**B**) 0–6 h.

The concentration versus time profile of sum total doxorubicin in plasma, brain tumor, peri-tumoral, and contralateral non-tumor brain after administration of NonL-doxo is presented in **Figure 1**. The pharmacokinetic parameters after administration of NonL-doxo are presented in **Table 1**. After administration of NonL-doxo, the plasma concentration versus time profile of sum total doxorubicin peaked at 0.083 h (5 min) after administration, and had a bi-phasic elimination profile as previously reported [12]. After administration of NonL-doxo, sum total doxorubicin concentrations were undetectable after 3 h in normal (contralateral, non-tumor) brain, 6 h in peri-tumoral brain, and 24 h in plasma and brain tumor. The exposure of sum total doxorubicin was higher in brain tumor compared to normal and peri-tumoral brain from 1 to 24 h.

**Table 1:** Pharmcokinetic parameters for PLD and NonL-doxo.

Matrix	Pharmacokinetic Parameters					
	PEGylated liposomal-doxorubicin			Non-liposomal doxorubicin		
	AUC_0-_(ng/mL·h)	t_max_(h)	C_max_(ng/mL)	AUC_0-∞_(ng/mL·h)	t_max_(h)	C_max_(ng/mL)
Plasma	2,257,480	3	197,020	1,545	0.083	831
Brain Tumor	229,716	3	2,181	12,134	6	570
Normal Brain	13,742	3	854	326	1	92
Peri-Tumoral Brain	23,972	3	725	283	3	32

Noncompartmental pharmacokinetic parameters following administration of PEGylated liposomal-doxorubicin and non-liposomal doxorubicin at 6 mg/kg IV ×1 in female athymic nude (nu/nu) mice bearing intracranial MDA-MB-231-BR human triple-negative breast cancer xenografts.

#### PEGylated liposomal-doxorubicin (PLD)

**Figure 2:**
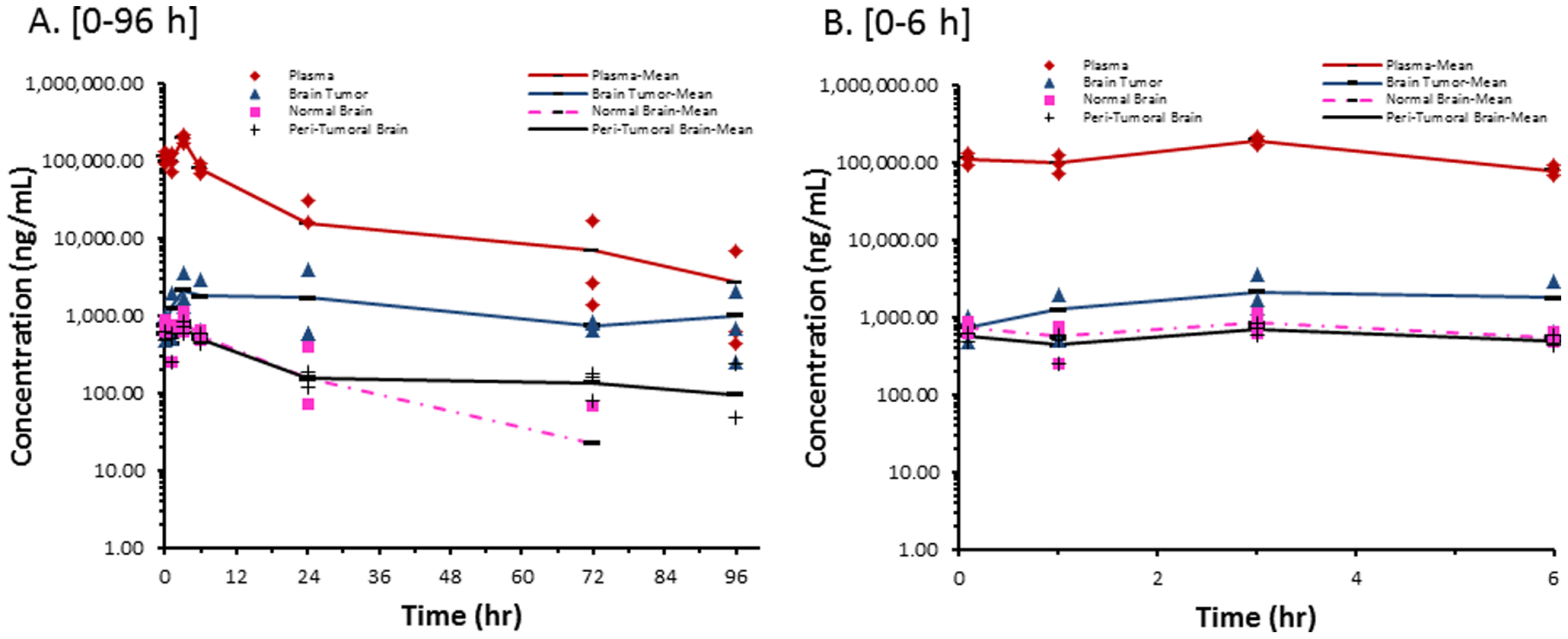
Sum Total Doxorubicin Concentrations from PLD. Individual and mean sum total doxorubicin concentration in plasma, brain tumor, contralateral non-tumor brain, and peri-tumoral brain of female athymic nude (nu/nu) mice bearing intracranial MDA-MB-231-BR human triple-negative breast cancer xenografts following administration of PEGylated liposomal-doxorubicin (PLD) at 6 mg/kg IV ×1. Samples (n = 3 mice at each time point) were obtained at 0.083, 1, 3, 6, 24, 72, and 96 hours following administration of PLD. (**A**) 0–96 h; (**B**) 0–6 h.

The concentration versus time profile of sum total doxorubicin in plasma, brain tumor, peri-tumoral brain and contralateral non-tumor brain after administration of PLD is presented in **Figure 2**. The pharmacokinetic parameters after administration of PLD are presented in **Table 1**. After administration of PLD, the plasma concentration versus time profile of sum total doxorubicin had a bi-phasic elimination profile, and was detectable until 96 h after administration. The long circulation of PLD in plasma was consistent with previous studies [25]. The concentration versus time profiles of sum total doxorubicin after administration of PLD in normal (contralateral, non-tumor) and peri-tumoral brain were similar to the profile in plasma; however, sum total doxorubicin concentration was undetectable in normal brain after 72 h. The exposure of sum total doxorubicin in tumor was higher than normal and peri-tumoral brain and was maintained until 96 h.

The plasma exposure for sum total doxorubicin as measured by the AUC was approximately 1,500-fold higher after administration of PLD as compared with NonL-doxo. CL and Vd of sum total doxorubicin after administration of PLD (CL: 2.65 mL/h/kg, Vd: 113.5 mL/kg, t_1/2_: 30 hours) were much lower as compared to those of NonL-doxo (CL: 3,882.7 mL/h/kg, Vd: 56,238 mL/kg, t_1/2_: 10 hours), indicating prolonged circulation and higher plasma exposures for PLD compared with NonL-doxo. The AUC of sum total doxorubicin after administration of PLD was approximately 20-fold higher in brain tumor, 42-fold higher in normal (contralateral non-tumor) brain, and 84-fold higher in peri-tumoral brain as compared with NonL-doxo, indicating greater delivery of sum total doxorubicin to brain tumor and brain.

### Single-agent efficacy results

**Figure 3:**
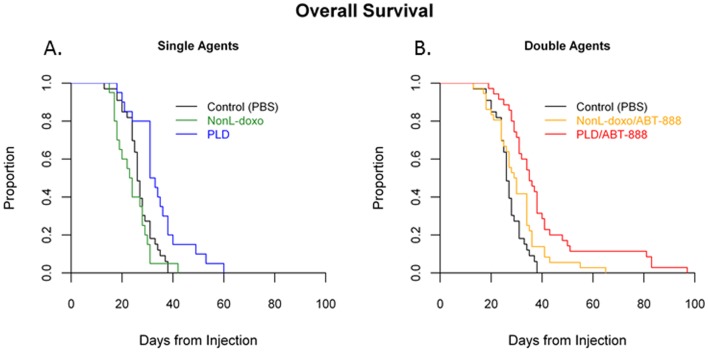
Efficacy Studies in an intracranial model of breast cancer. (**A**) Median survival (from the time of intracranial cell line injection) of animals treated with control (PBS, black), non-liposomal doxorubicin (NonL-doxo, green) 6mg/kg IV weekly and PEGylated liposomal doxorubicin (PLD, blue) 6mg/kg IV weekly in a murine model of intracranial breast cancer. (**B**) Median survival of animals treated with control (PBS, black), NonL-doxo 4.5 mg/mg IV weekly plus ABT-888 25 mg/kg OG daily (yellow) versus PLD (red) 4.5mg/mg IV weekly plus ABT-888 25 mg/kg OG daily in a murine model of intracranial breast cancer.

The therapeutic effect of NonL-doxo versus PLD was assessed in an intracranial model of breast cancer 7 to 14 days after stereotactic implantation of the MDA-MB-231-BR cells. Animals received weekly IV tail vein injections of either PBS control (100uL), NonL-doxo (6 mg/kg) or PLD (6mg/kg) until clinical symptoms necessitated sacrifice. The results are the product of 3 independent experiments across which there were no significant difference in the survival of control group animals (data not shown, [medians of 27 (CI = 24–31), 26 (CI = 18–28), 24 (CI = 18–29), p = 0.24]. The median survival of the control and NonL-doxo treated groups was not statistically different [p = 0.26; medians of 26 (CI = 25–28) and 23.5 days (CI 18–28), respectively; **Table 2** **;** **Figure 3A**]. In contrast, the median survival of PLD treated animals was 32 days (CI, 31–38) which was significantly longer than control (p = 0.0012) and NL- doxo (p = 0.0002) treated groups.

**Table 2:** Median survival of intracranial breast cancer model.

Treatment Groups	Dose(mg/kg)	Mice per group	Median Survival and 95% CI(days)	P value (compared to control)*
Control (PBS)	N/A	33	26 (25 – 28)	N/A
NonL-doxo	6mg/kg IV	20	23.5 (18 – 28)	0.26
PLD	6mg/kg IV	20	32 (31 – 38)	0.0012
ABT-888	25m/kg OG	18	27 (24 – 31)	0.5
NonL-doxo plus ABT-888	4.5mg/kg IV and 25mg/kg OG	36	29.5 (25 – 34)	0.04
PLD plus ABT-888	4.5mg/kg IV and 25mg/kg OG	35	35 (31 – 38)	< 0.0001

Median Survival of an intracranial breast cancer model treated with Control (PBS), non-liposomal doxorubicin (NonL-doxo) versus PEGylated Liposomal Doxorubicin (PLD).

* P value for NonL-doxo vs. PLD  = 0.0002; p value for NonL-doxo/ABT-888 vs. PLD/ABT-888 = 0.006.

Abbreviations: IV, intravenous; OG, oral gavage.

### PARP inhibitor combinations

#### Combination studies

To further augment therapeutic efficacy in the intracranial model of breast cancer, combination anthracycline-based therapy with the PARP inhibitor, ABT-888, was investigated. *In vitro* single agent and combination studies utilizing the MDA-MB-231-BR cell line were performed. As single agents, the IC50 doses of NonL-doxo and ABT-888 for the MDA-MB-231-BR cell line were 242 nM and 277 μM at 72 hours, respectively.

**Figure 4:**
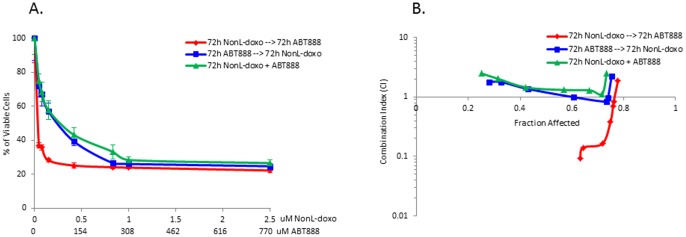
Sequential and combination treatment of the MDA-MB-231-BR cell line with ABT-888 and non-liposomal doxorubicin (NonL-doxo). (**A**) Percentage of viable cells treated in each of three treatment arms (72 hours [h] ABT-888 followed by 72 h NonL-doxo [blue], 72 h NonL-doxo followed by 72 h ABT-888 [red], and 72 h concurrent schedule of NonL-doxo and ABT-888 in combination [green]). (**B**) Combination index (CI) analysis in each arm compared to treatment with single agents. Note: CI <0.1–0.9, synergy; CI 0.9–1.1, additivity; CI >1.1, antagonism.

To determine if the combination of NonL-doxo and ABT-888 was additive, synergistic or antagonistic, MDA-MB-231-BR cells were treated with three schedules: 1) 72 hours NonL-doxo followed by 72 hours of ABT-888, 2) 72 hours ABT-888 followed by 72 hours NonL-doxo, and 3) a 72 hours of NonL-doxo and ABT-888 in combination. As shown in **Figure 4A**, treatment with NonL-doxo plus ABT-888 in combination for 72 hours showed similar results in terms of cell death compared to 72 h of ABT-888 prior to 72 h of NonL-doxo. However, sequencing NonL-doxo prior to ABT-888 resulted in enhanced cell death. Concordant with IC50 results and our hypothesis that DNA damage prior to inhibition of DNA repair would prove most effective, we observed that treatment with NonL-doxo for 72 h followed by ABT-888 for 72 h was synergistic at most doses (**Figure 4B**). Both combination treatment and sequential treatment with ABT-888 prior to NonL-doxo showed additivity and antagonism at most doses, with synergy at or above IC50 dosing.

In addition and in preparation for *in vivo* combination efficacy studies, a pilot toxicity study of NonL-doxo and ABT-888 was performed in 6 tumor-free FVB/NJ females aged 6 to 8 weeks (Jackson Laboratory, Stock #001800). Two mice were evaluated at each dose level of NonL-doxo: 6 mg/kg, 4.5 mg/kg, and 3 mg/kg (intraperitoneal) IP weekly ×3 weeks in combination with ABT-888 oral gavage (OG) 25 mg/kg/day. Treatment was administered for 21 days during which body mass and body composition score (BCS) were observed for a total of 49 days. Drug toxicity was noted in a dose-dependent manner. NonL-doxo 6mg/kg IP weekly with ABT-888 OG 25 mg/kg/day, was associated with early decline (both animals sacrificed at 30 days). NonL-doxo 4.5 mg/kg IP weekly in combination with ABT-888 OG 25 mg/kg/day resulted in one sacrifice (42 days); no other adverse effects were noted within the cohort. No adverse events were observed in the 3 mg/kg NonL-doxo plus ABT-888 mice throughout the study. Based on pilot dosing, historical data [16], [26], and the investigators' experience with PLD dosing, 4.5 mg/kg IV weekly for both NonL-doxo and PLD combined with ABT-888 25 mg/kg OG was selected as the maximum tolerated dose for further study.

#### Survival

Combination therapy of PLD with ABT-888 resulted in improved median overall survival (35 day, CI 31–38) as compared to control-treated (p<0.0001) and NonL-doxo plus ABT-888-treated animals (29.5 days, CI 25–34; p = 0.006; see **Table** **2** and **Figure** **3B**). Interestingly, the addition of ABT-888 to PLD only minimally improved the survival of animals treated with PLD alone (35 days, CI 31–38 versus 32 days, CI 31–38 days, respectively; p = 0.3; data not shown). Both therapies, PLD alone or with ABT-888 were superior to control-treated animals (p = 0.0012 and p<0.0001, respectively). Finally, and for completeness, single agent therapy with ABT-888 was compared to control with no differences observed (27 days, [CI 24–31] and 26 days [CI 25–28], p = 0.5; data not shown).

#### Bioluminescence studies

**Figure 5:**
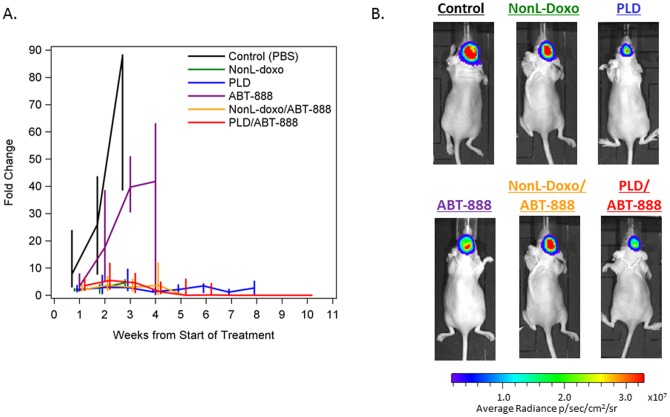
Bioluminescence imaging of TNBC intracranial tumor model. (**A**) Dynamic changes in intracranial tumor growth after treatment as measured by bioluminescence imaging in photons/second. Groups are as follows: Control (black), non-liposomal doxorubicin (NonL-doxo, green), PEGylated liposomal doxorubicin [PLD] (blue), ABT-888 (purple), NonL-doxo/ABT-888 (yellow) and PLD/ABT-888 (red). The median fold changes are connected over time for each treatment group. The vertical bars indicate the interquartile rages (25^th^–75^th^ percentiles). Points are only plotted when there were at least 2 animals in a treatment group. (**B**) Representative images of intracranial bioluminescence by treatment group 14 days post-treatment initiation.

In addition to overall survival, dynamic change (fold change from start of treatment as measured in photons/second) in the signal intensity of intracranial tumors was evaluated by bioluminescence imaging to assess efficacy of individual and combination therapeutics (**Figure** **5A and 5B**). As expected, the highest fold change from baseline in bioluminescence was observed in control-treated animals (median 87.8, Interquartile range [IQR] 38.5–88.5, 3 weeks post-treatment) followed by ABT-888-treated animals (median 41.8, IQR 0.03–63.2, 4 weeks post-treatment). The highest median fold-change in animals treated with either NonL-doxo or PLD as single agents was 4.6 (IQR 3.36–5.90, 3 weeks post-treatment) and 3.4 (IQR 0.79–4.37, 6 weeks post-treatment), respectively. Interestingly, among animals treated with combination NonL-doxo plus ABT-888 and PLD plus ABT-888, a peak in bioluminescence fold-change was noted early (median 3.67, IQR 1.58–7.04 and 5.42 IQR 2.01–11.95, respectively, 2 weeks post-treatment), followed by a decline in signal over time. At 5 weeks post-treatment, median bioluminescence fold-change was 0.10 (IQR 0.01–0.19) for NonL-doxo plus ABT-888-treated animals. Finally, 10 weeks post-treatment, median bioluminescence fold-change was 0.00 (IQR 0.00–0.00) for PLD plus ABT-888 animals.

## Discussion

As systemic therapies for breast cancer improve, the development of breast cancer brain metastases has emerged as a significant challenge in the management of patients with advanced breast cancer [1]. While the blood brain barrier is often compromised by the presence of intracranial tumor, it remains an impediment to systemic delivery of promising systemic therapies, including anthracyclines [27]. We report here, in a murine model of intracranial breast cancer, that PLD results in higher and prolonged plasma and intracranial tumor exposure as compared to NonL-doxo. Specifically, the sum total plasma AUC_0–∞_ of PLD was 1,500 times higher than that of NonL-doxo. Moreover, PLD was detected at 96 hours in plasma illustrating prolonged exposure as compared to NonL-doxo (undetectable after 24 hours). The plasma pharmacokinetic results of PLD and NonL-doxo are consistent with prior studies [10], [12], [13], [14], [28]. With regard to intracranial tumor, there were higher sum total doxorubicin concentrations after administration of PLD compared to NonL-doxo. Importantly, intracranial tumor sum total doxorubicin concentrations remained elevated at 96 hours, whereas the concentrations of doxorubicin after administration of NonL-doxo were non-detectable in brain and brain tumors after 24 hours.

In parallel to improved pharmacologic disposition, we report improved survival after administration of PLD as compared to control and NonL-doxo treatment in this MDA-MB-231-BR model of intracranial breast cancer. In contrast to our *in vitro* results, the addition of a small molecule PARP inhibitor capable of crossing the blood brain barrier, ABT-888[16], to PLD did not significantly improve survival as compared to single agent PLD. Interestingly, combination therapy with ABT-888 and PLD resulted in significant improvements in survival when compared to NonL-doxo plus ABT-888. In light of prior work illustrating similar efficacy and an improved toxicity profile (i.e. improved cardiac toxicity) for PLD as compared to NonL-doxo in patients with extracranial advanced breast cancer [29], our results provide sound preclinical rationale to support the design of clinical trials evaluating PLD with or without the PARP inhibitor, ABT-888, to treat patients with breast cancer brain metastases – particularly those with triple negative disease whose disease often presents both intra- and extracranially and for whom targeted systemic therapies are few [4]. However, while our results also support the study of other nanoparticle anticancer agents in the treatment of primary and metastatic intracranial tumors, we cannot recommend translation of our work to PLD with other PARP inhibitors, other than ABT-888, as the efficacy results of doxorubicin in combination with PARP inhibitors, (i.e. AG014699, INO-1001) have been mixed [30], [31].

The pharmacokinetics of nanoparticle drugs, e.g. PLD, is dependent upon the carrier and not the encapsulated drug until the drug is released from the carrier [32], [33], [34]. The drug that remains encapsulated within liposomes or nanoparticles is an inactive prodrug, thus the drug must be released from the carrier to be active (active warhead). Nanoparticles and liposomes are cleared via the mononuclear phagocyte system (MPS), which is located primarily in the liver and spleen, as well as in the lung, bone marrow and blood [28], [35], [36], [37]. Nanoparticles and liposomes can alter both the tissue distribution and the clearance of drugs because the drug takes on the PK characteristics of the carrier [10], [33], [34]. The ability of nanoparticle agents to deliver drug to brain/brain tumors has not been extensively evaluated. However, our study suggests that PLD achieved approximately 20-fold higher exposures of drug into the brain tumors than the NonL-doxo. Moreover, the results of this study can have a far reaching impact as there are >300 nanoparticle anticancer agents containing various anticancer agents as cargo in preclinical and clinical development that may have enhanced delivery to intracranial tumors, which may results in greater efficacy for the treatment of CNS-located epithelial malignancies [33], [34], [38].

Prior studies also suggest that nanoparticle agents can deliver more drug to brain than their non-nanoparticle counterparts. Walsh et al [11] showed that XMT-1001, a macromolecular camptothecin (CPT) conjugate, administered at 60mg CPT equivalents/kg in mice bearing HT-29 human colon carcinoma xenografts delivered 5-fold higher exposures of camptothecin into brain than irinotecan (administered at 100 mg/kg CPT equivalents). In a study comparing the plasma, tumor, and tissue pharmacokinetics of PEGylated liposomal CKD-602 (S-CKD602) and nonliposomal CKD-602 (a camptothecin analog) in mice bearing A-375 human melanoma xenografts, S-CKD602 delivered approximately 3-fold higher exposures of the CKD-602 into brain as compared to non-liposomal CKD-602 [10] after administration of only 1/30^th^ of the dose of non-liposomal CKD-602. Siegal et al. showed that PLD achieved 15-fold higher doxorubicin levels in brain tumors of fisher rats after 48 hours of administration as compared to NonL-doxo [12].

Although the mechanism of enhanced CNS delivery of nanoparticles is not completely understood, it is postulated that higher exposure to CNS tumors is related to nanoparticle longevity in circulation compared to non-nanoparticle formulations. Penetration of small molecule anti-cancer agents across the blood brain barrier is limited by molecular weight, polarity, efflux mechanisms (i.e. P-glycoprotein), and short half-life [8], [9]. We hypothesize that prolonged systemic exposure afforded by nanoparticle technology allows for permeation of tumor microcirculation via passive convection transport through a BBB potentially “compromised” by the presence of tumor. Chances for extravasation improve with prolonged circulation half-life and a greater number of circulation passages through a tumor bed. Although factors inherent to intracranial tumor (i.e. lack of lymphatic drainage, increased intracranial pressure) may dampen the effect of nanoparticle transport into a tumor compartment, results of preclinical and clinical studies argue that longer circulation time afforded by nanoparticle formulations abrogate these effects [12], [13], [14]. Moreover, several investigators have sought to augment nanoparticle delivery to the CNS via strategic targeting of liposomes to receptors selectively expressed by cell lines and human tumors. As an example, treatment with doxorubicin encapsulated in interleukin-13 (IL-13)-conjugated liposomes resulted in improved survival and enhanced tumor reduction in an intracranial model of glioblastoma multiforme (GBM) as compared to unconjugated liposomes with the same doxorubicin concentration [39]. More specific to breast cancer, a second study has shown that PLD targeting integrin α5β1, over-expressed in tumor vasculature and cancer cells, results in higher cytotoxicity when treating MDA-MB-231 α5β1-expressing, breast cancer cell lines than non-targeted liposomes [40]. Future studies of targeted liposomes to treat intracranial breast cancer are warranted.

We recognize that our study has several limitations. First, the murine model selected to perform both the pharmacokinetic and efficacy studies of PLD versus NonL-doxo is an intracranial model of breast cancer per direct intracranial implantation. The goal of this project; however, was to treat established intracranial tumor to test intracranial drug delivery and efficacy as opposed to prevention of colonization of the central nervous system pharmacologically (i.e. tumor growth inhibition) in which models of brain metastases arising from hematogenous injection are routinely used [27]. Moreover, an intracranial approach has historically been employed as an accepted model to study both secondary and primary brain tumors [12], [41]. We view our data as a “proof of concept” such that other pharmacologic agents (both chemotherapeutics and targeted agents) may be more efficiently delivered to the central nervous system using similar technology.

## Conclusions

In conclusion, results of this study indicate that the pharmacologic and efficacy profile of PLD is superior to that of NonL-doxo in an intracranial model of established breast cancer brain metastases. Moreover, the addition of the PARP inhibitor, ABT-888, to PLD resulted in improved survival as compared to NonL-doxo plus ABT-888 in this model. Taken together, our results represent a novel and efficacious strategy in a preclinical setting – liposomal delivery of a chemotherapeutic alone or in combination with a small molecule PARP inhibitor – to treat breast cancer brain metastases. These results provide strong rationale to translate our findings into early phase trials evaluating PLD, with or without ABT-888, among patients with breast cancer brain metastases, including those with triple negative disease, with the goal of improving outcome for patients with such a devastating disease.
